# Laboratory-Confirmed COVID-19–Associated Hospitalizations Among Adults During SARS-CoV-2 Omicron BA.2 Variant Predominance — COVID-19–Associated Hospitalization Surveillance Network, 14 States, June 20, 2021–May 31, 2022

**DOI:** 10.15585/mmwr.mm7134a3

**Published:** 2022-08-26

**Authors:** Fiona P. Havers, Kadam Patel, Michael Whitaker, Jennifer Milucky, Arthur Reingold, Isaac Armistead, James Meek, Evan J. Anderson, Andy Weigel, Libby Reeg, Scott Seys, Susan L. Ropp, Nancy Spina, Christina B. Felsen, Nancy E. Moran, Melissa Sutton, H. Keipp Talbot, Andrea George, Christopher A. Taylor, COVID-NET Surveillance Team, COVID-NET Surveillance Team, Pam Daily Kirley, Nisha B. Alden, Kimberly Yousey-Hindes, Kyle P. Openo, Chloe Brown, Cody T. Schardin, Kelly Plymesser, Grant Barney, Kevin Popham, Laurie M. Billing, Nasreen Abdullah, Tiffanie M. Markus, Mary Hill

**Affiliations:** ^1^CDC COVID-19 Emergency Response Team; ^2^General Dynamics Information Technology, Atlanta, Georgia; ^3^University of California, Berkeley, Berkeley, California; ^4^Colorado Department of Public Health and Environment; ^5^Connecticut Emerging Infections Program, Yale School of Public Health, New Haven, Connecticut; ^6^Emory University School of Medicine, Atlanta, Georgia; ^7^Georgia Emerging Infections Program, Georgia Department of Public Health; ^8^Atlanta Veterans Affairs Medical Center, Atlanta, Georgia; ^9^Iowa Department of Public Health; ^10^Michigan Department of Health and Human Services; ^11^Minnesota Department of Health; ^12^New Mexico Department of Health; ^13^New York State Department of Health; ^14^University of Rochester School of Medicine and Dentistry, Rochester, New York; ^15^Ohio Department of Health; ^16^Public Health Division, Oregon Health Authority; ^17^Vanderbilt University Medical Center, Nashville, Tennessee; ^18^Salt Lake County Health Department, Utah.; California Emerging Infections Program, Oakland, California; Colorado Department of Public Health and Environment; Connecticut Emerging Infections Program, Yale School of Public Health, New Haven, Connecticut; Emory University School of Medicine, Georgia Emerging Infections Program, Georgia Department of Public Health, Atlanta Veterans Affairs Medical Center, Atlanta, Georgia; Michigan Department of Health and Human Services, Lansing, Michigan; Minnesota Department of Health; New Mexico Department of Health; New York State Department of Health; University of Rochester School of Medicine and Dentistry, Rochester, New York; Ohio Department of Health; Public Health Division, Oregon Health Authority; Vanderbilt University Medical Center, Nashville, Tennessee; , Salt Lake County Health Department, Utah.

Beginning the week of March 20–26, 2022, the Omicron BA.2 variant of SARS-CoV-2, the virus that causes COVID-19, became the predominant circulating variant in the United States, accounting for >50% of sequenced isolates.* Data from the COVID-19–Associated Hospitalization Surveillance Network (COVID-NET) were analyzed to describe recent COVID-19–associated hospitalization rates among adults aged ≥18 years during the period coinciding with BA.2 predominance (BA.2 period [Omicron BA.2 and BA.2.12.1; March 20–May 31, 2022]). Weekly hospitalization rates (hospitalizations per 100,000 population) among adults aged ≥65 years increased threefold, from 6.9 (week ending April 2, 2022) to 27.6 (week ending May 28, 2022); hospitalization rates in adults aged 18–49 and 50–64 years both increased 1.7-fold during the same time interval. Hospitalization rates among unvaccinated adults were 3.4 times as high as those among vaccinated adults. Among hospitalized nonpregnant patients in this same period, 39.1% had received a primary vaccination series and 1 booster or additional dose; 5.0% had received a primary series and ≥2 boosters or additional doses. All adults should stay up to date^†^ with COVID-19 vaccination, and multiple nonpharmaceutical and medical prevention measures should be used to protect those at high risk for severe COVID-19 illness, irrespective of vaccination status^§^ (*1*).

COVID-NET conducts population-based surveillance for laboratory-confirmed COVID-19–associated hospitalizations (defined as receipt of a positive SARS-CoV-2 molecular or rapid antigen detection test result during hospitalization or during the 14 days preceding admission) in 99 counties across 14 U.S. states.^¶^ This analysis describes weekly hospitalization rates among adults aged ≥18 years during June 20, 2021–May 28, 2022; monthly clinical and vaccination data were available through May 31, 2022. Data from the BA.2 period were compared with those from the Delta (B.1.617.2; June 20–December 18, 2021) and BA.1 (Omicron B.1.1.529 and BA.1.1; December 19, 2021–March 19, 2022) periods.

Among all adults,** hospitalization rates were calculated overall, and by age and COVID-19 vaccination status. Vaccination status (i.e., unvaccinated, received primary series only, or received primary series and ≥1 booster or additional dose) was determined for individual hospitalized patients and for the catchment population using state immunization information systems data. Recipients of primary series only include hospitalized persons who received a positive SARS-CoV-2 test result from a specimen collected ≥14 days after either the second of a 2-dose vaccination series or after 1 dose of a single-dose vaccine but who have not received a booster or additional doses. Recipients of primary series with ≥1 booster or additional dose include hospitalized persons who received a primary vaccination series and a booster or additional dose on or after August 13, 2021, with a positive SARS-CoV-2 test result from a specimen collected ≥14 days after receipt of ≥1 booster or additional dose. Because the immune status of all patients is not known, an additional dose (recommended for persons with a compromised immune system) cannot be distinguished from a booster dose. This issue is a relevant consideration because vaccines can be less effective in persons with a compromised immune system. These data do not yet distinguish between multiple booster or additional doses. Unvaccinated patients include those with a positive SARS-CoV-2 test result who have no record of receiving any COVID-19 vaccine doses^††^ (*2*). Rate ratios were calculated by dividing rates of hospitalization among unvaccinated persons by rates among vaccinated persons by month and by period of variant predominance and age group.

Using previously described methods (*3*,*4*), clinical data were collected on an age- and site-stratified representative sample of hospitalized adult patients. Pregnant patients were excluded because their reasons for hospital admission (*3*) might differ from those for nonpregnant persons. Surveillance officers abstracted data on sampled patients from medical charts, including reason for admission.^§§^

Percentages presented were weighted to account for the probability of selection for sampled cases. Variances were estimated using Taylor series linearization method. Analyses were conducted using SAS statistical software survey procedures (version 9.4; SAS Institute). This activity was reviewed by CDC and conducted consistent with applicable federal law and CDC policy.^¶¶^

During June 20, 2021–May 31, 2022, a total of 121,007 hospitalizations in COVID-NET were recorded. During the BA.2 period, among adults aged ≥65 years, hospitalization rates increased threefold, from a nadir of 6.9 (week ending April 2, 2022) to a peak of 27.6 (week ending May 28, 2022). During the same weeks, rates increased from 1.3 to 3.6 among adults aged 18–49 years and from 2.7 to 7.4 among adults aged 50–64 years, both a 1.7-fold increase (Figure 1). Compared with adults aged 18–49 years, hospitalization rate ratios for adults aged ≥65 years were 3.9, 5.7, and 8.2 in the Delta, BA.1, and BA.2 periods, respectively (Figure 2).

**FIGURE 1:**
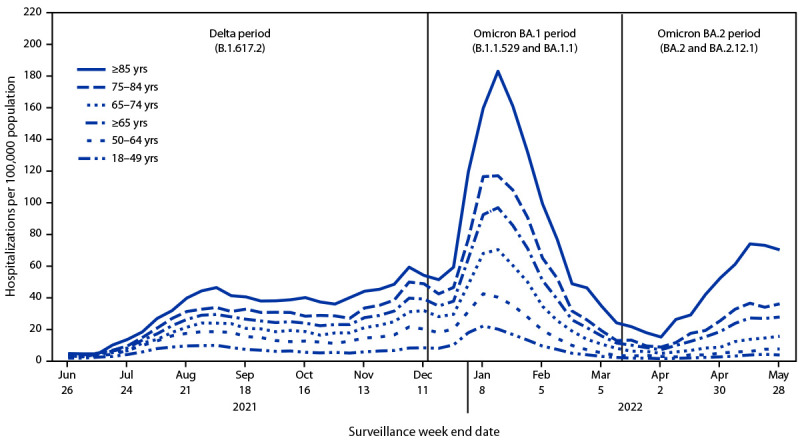
Weekly COVID-19-associated hospitalization rates among adults aged ≥18 years, by age group and period of COVID-19 variant predominance* — COVID-19–Associated Hospitalization Surveillance Network, 14 states,^†^ weeks ending June 26, 2021–May 28, 2022^§^ * SARS-CoV-2 variant predominance defined by period when variant accounted for >50% of sequenced isolates. † Data are collected in selected counties in California, Colorado, Connecticut, Georgia, Iowa, Maryland, Michigan, Minnesota, New Mexico, New York, Ohio, Oregon, Tennessee, and Utah. A list of these counties is available at https://www.cdc.gov/mmwr/volumes/69/wr/mm6915e3.htm. Iowa did not provide immunization data but is included in the overall population-based hospitalization rates. Additional information on surveillance methods is available at https://www.cdc.gov/coronavirus/2019-ncov/covid-data/covid-net/purpose-methods.html. ^§^ Maryland did not contribute data after December 4, 2021, but did contribute data for previous weeks.

**FIGURE 2:**
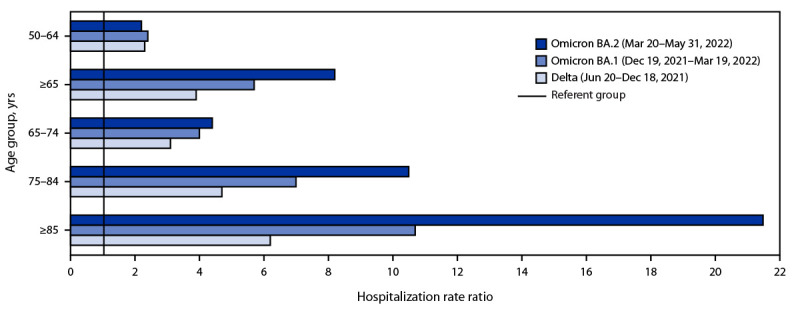
COVID-19–associated hospitalization rate ratios* among adults aged ≥18 years, by age group and period of COVID-19 variant predominance — COVID-19–Associated Hospitalization Surveillance Network, 14 states,^†^ July 2021–May 2022^§^ * Adults aged 18–49 years are the referent group; the rate ratio for this group is 1.0 for all periods. ^†^ Data are collected in selected counties in California, Colorado, Connecticut, Georgia, Iowa, Maryland, Michigan, Minnesota, New Mexico, New York, Ohio, Oregon, Tennessee, and Utah. A list of these counties is available at https://www.cdc.gov/mmwr/volumes/69/wr/mm6915e3.htm. Iowa did not provide immunization data but is included in the overall population-based hospitalization rates. Additional information on surveillance methods is available at https://www.cdc.gov/coronavirus/2019-ncov/covid-data/covid-net/purpose-methods.html. ^§^ Maryland did not contribute data after December 4, 2021, but did contribute data for previous weeks.

Among 8,266 nonpregnant adults whose medical charts were abstracted, adults aged ≥65 years accounted for 41.0%, 49.6% and 61.5% of hospitalizations during the Delta, BA.1, and BA.2 periods, respectively (Table). Among nonpregnant hospitalized adults who received a positive SARS-CoV-2 test result, the proportion likely admitted for a COVID-19–related illness accounted for 95.5% of hospitalizations during the Delta period, declining to 87.8% and 85.4% in the BA.1 and BA.2 periods, respectively. In adults aged ≥65 years, the proportions likely admitted for a COVID-19–related illness were 96.4%, 92.6%, and 93.4% for the Delta, BA.1, and BA.2 periods, respectively; these proportions were 95.8%, 89.2%, and 80.8% in adults aged 50–64 years and 93.4%, 76.3% and 70.6% in adults aged 18–49 years, respectively.

**TABLE:** Demographic characteristics and clinical interventions and outcomes among nonpregnant adults aged ≥18 years hospitalized with COVID-19* during periods of SARS-CoV-2 B.1.617.2 (Delta), Omicron BA.1, and Omicron BA.2 predominance^†^ (N = 8,266) — COVID-19–Associated Hospitalization Surveillance Network, 14 states,^§^ June 20, 2021–May 31, 2022^¶^

Characteristic	Hospitalizations,** No. (%)		
	Delta	Omicron BA.1	Omicron BA.2
	(Jun 20–Dec 18, 2021)	(Dec 19, 2021–Mar 19, 2022)	(Mar 20–May 31, 2022)
**Overall**	**5,234 (52.2)**	**1,804 (38.5)**	**1,228 (9.2)**
**Demographic characteristic^††^**			
**Age, median, yrs (IQR)**	59.9 (46.7–72.0)	63.8 (49.8–76.8)	70.5 (55.8–81.5)
**Age group, yrs**			
18–49	1,523 (28.5)	501 (24.1)	312 (17.5)
50–64	1,859 (30.5)	615 (26.3)	480 (21.0)
≥65	1,852 (41.0)	688 (49.6)	436 (61.5)
65–74	859 (19.2)	287 (19.6)	136 (18.2)
75–84	635 (14.0)	248 (17.5)	175 (24.2)
≥85	358 (7.8)	153 (12.6)	125 (19.1)
**Sex**			
Male	2,782 (52.9)	971 (52.5)	635 (51.0)
Female	2,452 (47.1)	833 (47.5)	593 (49.0)
**Race and ethnicity^§§^**			
White	3,138 (58.0)	1,103 (55.2)	811 (69.5)
Black or African American	1,012 (23.7)	319 (26.2)	208 (15.7)
AI/AN	67 (1.5)	21 (1.3)	13 (0.6)
A/PI	144 (3.5)	51 (4.6)	46 (7.2)
Hispanic or Latino	652 (13.3)	219 (12.7)	118 (7.0)
**Long-term care facility residence^¶¶^**	289 (5.7)	146 (9.0)	134 (14.2)
**Any underlying medical condition**	4,556 (89.3)	1,596 (91.7)	1,118 (95.1)
**Immunosuppressive condition**	535 (11.0)	288 (16.0)	225 (19.2)
Reason for admission***			
Likely COVID-19–related	4,838 (95.5)	1,530 (87.8)	1,009 (85.4)
Inpatient surgery	43 (0.4)	44 (1.9)	49 (3.2)
Psychiatric admission requiring medical care	80 (1.4)	72 (3.8)	61 (4.2)
Trauma	78 (1.2)	63 (3.1)	49 (3.2)
Other	72 (1.3)	48 (3.1)	37 (3.7)
Unknown	14 (0.2)	7 (0.4)	6 (0.4)
**Vaccination status** ^†††^			
Unvaccinated	3,516 (69.4)	800 (47.2)	377 (27.8)
Primary series	1,269 (25.1)	551 (32.6)	322 (24.3)
Primary series with ≥1 booster or additional dose	48 (1.4)	310 (15.6)	443 (44.1)
Primary series with 1 booster or additional dose	43 (1.3)	297 (14.9)	398 (39.1)
Primary series with ≥2 boosters or additional doses	5 (0.1)	13 (0.7)	45 (5.0)
**Length of hospital stay, days, median (IQR)**	4.8 (2.4–10.0)	3.9 (1.9–8.7)	3.3 (1.6–7.4)
**ICU admission^§§§^**	1,252 (24.3)	338 (17.9)	187 (13.2)
**Received mechanical ventilation^¶¶¶^**	676 (13.5)	153 (7.6)	80 (5.7)
In-hospital death****	574 (12.4)	131 (7.5)	48 (5.1)

**Abbreviations:** AI/AN = American Indian or Alaska Native; A/PI = Asian or Pacific Islander; COVID-NET = COVID-19–Associated Hospitalization Surveillance Network; ICU = intensive care unit.

* Data are from a weighted sample of hospitalized nonpregnant adults with completed medical record abstractions and a discharge disposition. Sample sizes presented are unweighted with weighted percentages.

^†^ During the Delta period (June 20–December 18, 2021), the Delta (B.1.617.2) variant was the predominant variant (accounting for >50% of sequenced case isolates) in the United States. For the B.1 period (December 19, 2021–March 19, 2022), B.1.1.529 and BA.1.1 were the predominant Omicron variants. For the BA.2 period (March 20, 2022–May 31, 2022), the predominant variants were Omicron subvariants BA.2 and BA.2.12.1.

^§^ Data are collected in selected counties in California, Colorado, Connecticut, Georgia, Iowa, Maryland, Michigan, Minnesota, New Mexico, New York, Ohio, Oregon, Tennessee, and Utah. A list of these counties is available at https://www.cdc.gov/mmwr/volumes/69/wr/mm6915e3.htm. Iowa did not provide immunization data but is included in the overall population-based hospitalization rates. Additional information on surveillance methods is available at https://www.cdc.gov/coronavirus/2019-ncov/covid-data/covid-net/purpose-methods.html.

^¶^ Maryland did not contribute data after December 4, 2021, but did contribute data for previous weeks.

** Total hospitalizations include data from selected counties in all 14 COVID-NET states with vaccination status, including fully vaccinated, partially vaccinated, and unvaccinated adults. As a result, the number of total hospitalizations exceeds the sum of fully vaccinated and unvaccinated adults.

^††^ Percentages presented for demographic and other characteristics are weighted column percentages.

^§§^ Black or African American, White, AI/AN, and A/PI persons were not Hispanic or Latino (non-Hispanic); Hispanic or Latino (Hispanic) persons could be of any race. If Hispanic ethnicity was unknown, non-Hispanic ethnicity was assumed. Persons with multiple, unknown, or missing race accounted for 3.4% (weighted) of all cases. These persons are excluded from the proportions of race and ethnicity but are otherwise included elsewhere in the analysis.

^¶¶^ Long-term care facility residents include hospitalized adults who were identified as residents of a nursing home or skilled nursing facility, rehabilitation facility, assisted living or residential care, long-term acute care hospital, group or retirement home, or other long-term care facility upon hospital admission. A free-text field for other types of residences was examined; patients with a long-term care facility-type residence were also categorized as long-term care facility residents.

*** COVID-19–related illness as a likely reason for admission is indicated by COVID-19 diagnosis or symptoms consistent with COVID-19 as the chief complaint or reason for admission in the history of present illness. COVID-19–related symptoms included respiratory signs and symptoms (e.g., congestion or runny nose, cough, hemoptysis or bloody sputum, shortness of breath or respiratory distress, sore throat, upper respiratory infection, influenza-like illness, and wheezing) and nonrespiratory signs and symptoms (e.g., abdominal pain, altered mental status or confusion, anosmia or decreased smell, chest pain, conjunctivitis, diarrhea, dysgeusia or decreased taste, fatigue, fever or chills, headache, muscle aches or myalgias, nausea or vomiting, rash, and seizures). Non–COVID-19 reason for admissions included planned inpatient surgery or procedures, psychiatric admission needing acute medical care, trauma, other, and unknown. Two physicians reviewed other reasons for admission and chief complaints to determine whether they likely were not COVID-19-related (e.g., skin and soft tissue infections).

^†††^ Primary series only includes persons who received a positive SARS-CoV-2 test result from a specimen collected ≥14 days after either the second of a 2-dose vaccination series or after 1 dose of a single dose vaccine but no booster or additional doses. Primary series with ≥1 booster or additional dose includes persons who received a primary vaccination series and a booster or additional dose on or after August 13, 2021, with a positive SARS-CoV-2 test result from a specimen collected ≥14 days after receipt of ≥1 booster or additional dose. Persons who did not receive any COVID-19 vaccine dose were considered unvaccinated. Partially vaccinated persons who received ≥1 vaccine dose but did not complete a primary series ≥14 days before a positive SARS-CoV-2 test result are excluded from data shown.

**^§§§^** ICU admission status was missing in 1.3% (weighted) of hospitalizations; these hospitalizations are otherwise included elsewhere in the analysis.

**^¶¶¶^** Invasive mechanical ventilation status was missing in 1.4% (weighted) of hospitalizations; these hospitalizations are otherwise included elsewhere in the analysis.

**** In-hospital death status was missing in 1.3% (weighted) of hospitalizations; these hospitalizations are otherwise included elsewhere in the analysis.

During the BA.2 period, 27.8% of hospitalized adults were unvaccinated, representing a 60% decrease from 69.4% during the Delta period and a 41% decrease from 47.2% during the BA.1 period. The proportion who had received a primary series and ≥1 booster or additional dose increased from 1.4%, 15.6%, and 44.1% during the Delta, BA.1, and BA.2 periods, respectively. In May 2022, the monthly population-based, age-adjusted hospitalization rate among unvaccinated adults aged ≥18 years was 3.4 times as high (95% CI = 3.2–3.6) as rates among vaccinated adults who had received ≥1 booster or additional dose (CDC, COVID-19–Associated Hospitalization Surveillance Network, unpublished data, 2022).

During all periods, the percentage of hospitalized adults with at least one underlying medical condition ranged from 89.3% (Delta) to 95.1% (BA.2). Proportions of hospitalized adults admitted to an intensive care unit during the Delta, BA.1 and BA.2 periods were 24.3%, 17.9% and 13.2%, respectively. The proportion of in-hospital deaths during these periods declined from 12.4% (Delta) to 7.5% (BA.1) and 5.1% (BA.2).

## Discussion

During March 20–May 31, 2022, coinciding with the period of the Omicron BA.2 variant predominance, COVID-19–associated hospitalization rates increased among adults aged ≥65 years relative to those in younger adults, and a higher proportion of those hospitalized were aged ≥65 years compared with that during the Delta and BA.1 periods. Nearly all hospitalized adults had one or more underlying medical condition. Hospitalization rates continue to remain higher among unvaccinated adults than among adults who received a primary COVID-19 vaccination series and ≥1 booster or additional dose. Approximately one third of hospitalized adults during the BA.2 period completed a primary series and received 1 booster or additional dose, and 5.0% received ≥2 booster or additional doses. These findings underscore the continued risk for COVID-19–associated hospitalization, particularly among unvaccinated persons and among older adults, irrespective of vaccination status.

Older adults have experienced the highest hospitalization rates throughout the COVID-19 pandemic, and the proportion of hospitalized adults aged ≥65 years increased during the Delta and Omicron periods. Approximately 90% of COVID-NET hospitalizations among adults aged ≥65 years during the BA.2 period were likely admitted for COVID-19–related illness, which demonstrates that severe COVID-19 continues to affect older adults.

Multiple reasons likely contribute to the disproportionate increase in COVID-19–associated hospitalization rates among older adults. Older age remains the strongest risk factor for severe COVID-19 outcomes; other risk factors include the presence of certain underlying medical conditions*** and being unvaccinated or not having received a COVID-19 primary vaccination series and a booster dose. Although vaccines remain effective at preventing severe illness (*5*), the proportion of hospitalized patients who are vaccinated is expected to increase as vaccination coverage increases. As of July 6, 2022, 91.6% of adults aged ≥65 years had received a primary series, 64.4% had received 1 booster or additional dose, and 22.2% received a second booster or additional doses,^†††^ which was recommended for adults aged ≥50 years on March 29, 2022, during the BA.2 period.^§§§^ Not being up to date with COVID-19 vaccination might contribute to the increased hospitalization rates among adults in this age group. In addition, COVID-19 vaccine effectiveness has been found to decline 6 months after vaccination, at least in part because of waning immunity, which might disproportionately affect rates among vaccinated older adults who received approval for vaccines earlier than did those in other age groups^¶¶¶^ (*6*).

Nearly one half of adults hospitalized during the BA.2 period had received a primary vaccination series and ≥1 booster or additional dose. This finding indicates that in addition to increasing vaccination coverage and encouraging all adults to stay up to date with vaccinations, other multiple nonpharmaceutical and medical prevention measures should be implemented to protect persons at high risk for severe illness and hospitalization because of older age, disability, moderate or severe immunocompromise, or other underlying medical conditions (*1*). These additional measures include the use of masks or respirators that provide more protection for the wearer,**** early access to and use of antivirals, including ritonavir-boosted nirmatrelvir (Paxlovid) and remdesivir (Veklury),^††††^ preexposure prophylaxis if indicated (e.g., Evusheld for persons who are immunocompromised), and following guidance on testing, isolation, and managing exposures^§§§§^ (*1*).

The findings in this report are subject to at least five limitations. First, some COVID-19–associated hospitalizations might have been missed because of hospital testing practices. Second, vaccination status is subject to misclassification, which might affect estimation of rates by vaccination status. In addition, because immunocompromise status is not always known, it is not possible to distinguish between booster and additional doses administered to persons who are immunocompromised; not having this information could also have influenced observed rates. Third, information on prehospital COVID-19 treatment was not reliably available in abstracted inpatient records to aid interpretation of clinical data. Fourth, the reason for admission was determined based on a specified algorithm; misclassification might have occurred, because reasons for admission are not always clear. Even among hospitalizations in which COVID-19 was not a likely reason for admission, COVID-19 might still affect clinical decision-making and outcomes. Finally, COVID-NET catchment areas include approximately 10% of the U.S. population; thus, findings might not be nationally generalizable.

Coinciding with the predominance of the Omicron BA.2 variant, COVID-19–associated hospitalization rates increased during March–May 2022, mainly among adults aged ≥65 years. Hospitalization rates continue to be higher among those who are unvaccinated compared with those who were vaccinated with a primary series and ≥1 booster or additional dose. Older adults and those with underlying medical conditions, including those who have been vaccinated, might still be at risk for severe disease as demonstrated by the fact that nearly one half of hospitalized patients during the BA.2 period had received a primary series and ≥1 booster or additional dose. In addition to staying up to date with vaccinations, other multiple nonpharmaceutical and medical prevention measures are important to reduce the risk for hospitalization among adults at high risk for severe COVID-19 illness.

**Summary**

**What is already known about this topic?**

Older adults and those with underlying medical conditions infected with SARS-CoV-2 have increased risks for hospitalization.

**What is added by this report?**

Increased hospitalization rates among adults aged ≥65 years compared with rates among younger adults were most pronounced during the Omicron BA.2–predominant period. Among hospitalized nonpregnant patients, 44.1% had received primary vaccination and ≥1 booster or additional dose. Hospitalization rates among unvaccinated adults were approximately triple those of vaccinated adults.

**What are the implications for public health practice?**

Adults should stay up to date with COVID-19 vaccination, including booster doses. Multiple nonpharmaceutical and medical prevention measures should be used to protect persons at high risk for severe SARS-CoV-2, regardless of vaccination status.

## Footnotes

* https://data.cdc.gov/Laboratory-Surveillance/SARS-CoV-2-Variant-Proportions/jr58-6ysp (Accessed August 21, 2022).

†
https://www.cdc.gov/coronavirus/2019-ncov/vaccines/stay-up-to-date.html

§
https://www.cdc.gov/coronavirus/2019-ncov/your-health/treatments-for-severe-illness.html

¶ Data are collected in selected counties in California, Colorado, Connecticut, Georgia, Iowa, Maryland, Michigan, Minnesota, New Mexico, New York, Ohio, Oregon, Tennessee, and Utah. A list of these counties is available at https://www.cdc.gov/mmwr/volumes/69/wr/mm6915e3.htm. Iowa did not provide immunization data but is included in the overall population-based hospitalization rates. Maryland did not contribute data after December 4, 2021, but did contribute data for previous weeks. Additional information on surveillance methods is available at https://www.cdc.gov/coronavirus/2019-ncov/covid-data/covid-net/purpose-methods.html.

** Rates are calculated using the CDC National Center for Health Statistics’ vintage 2020 bridged-race postcensal population estimates for the counties included in surveillance (https://www.cdc.gov/nchs/nvss/bridged_race.htm). Rates cannot be stratified by pregnancy status because the underlying population of pregnant persons in the catchment area is unknown.

†† https://covid.cdc.gov/covid-data-tracker/#covidnet-hospitalizations-vaccination (Accessed August 21, 2022).

§§ COVID-19–related illness as a likely reason for admission is indicated by COVID-19 diagnosis or symptoms consistent with COVID-19 as the chief complaint or reason for admission in the history of present illness. COVID-19–related signs and symptoms included respiratory symptoms (e.g., congestion or runny nose, cough, hemoptysis or bloody sputum, shortness of breath or respiratory distress, sore throat, upper respiratory infection, influenza-like illness, and wheezing) and nonrespiratory signs and symptoms (e.g., abdominal pain, altered mental status or confusion, anosmia or decreased smell, chest pain, conjunctivitis, diarrhea, dysgeusia or decreased taste, fatigue, fever or chills, headache, muscle aches or myalgias, nausea or vomiting, rash, and seizures). Non–COVID-19 reasons for admission included planned inpatient surgery or procedures, psychiatric admission needing acute medical care, trauma, other, and unknown. Two physicians reviewed other reasons for admission and chief complaints to determine whether they likely were not COVID-19–related (e.g., skin and soft tissue infections).

¶¶ 45 C.F.R. part 46, 21 C.F.R. part 56; 42 U.S.C. Sect. 241(d); 5 U.S.C. Sect. 552a; 44 U.S.C. Sect. 3501 et seq.

***
https://www.cdc.gov/coronavirus/2019-ncov/hcp/clinical-care/underlyingconditions.html

††† https://data.cdc.gov/Vaccinations/COVID-19-Vaccinations-in-the-United-States-Jurisdi/unsk-b7fc/data (Accessed August 21, 2022).

§§§
https://www.cdc.gov/media/releases/2022/s0328-covid-19-boosters.html

¶¶¶
https://www.cdc.gov/vaccines/hcp/acip-recs/vacc-specific/covid-19.html

**** https://www.cdc.gov/coronavirus/2019-ncov/prevent-getting-sick/types-of-masks.html#DifferentSituations ^††††^ https://www.cdc.gov/coronavirus/2019-ncov/hcp/clinical-care/outpatient-treatment-overview.html; https://www.covid19treatmentguidelines.nih.gov/management/clinical-management/clinical-management-summary/

§§§§ https://www.fda.gov/media/154703/download; https://www.covid19treatmentguidelines.nih.gov/overview/prioritization-of-therapeutics/

## References

[1] Massetti GM, Jackson BR, Brooks JT, Summary of guidance for minimizing the impact of COVID-19 on individual persons, communities, and health care systems—United States, August 2022. MMWR Morb Mortal Wkly Rep 2022;71:1057–64. 10.15585/mmwr.mm7133e1 35980866 PMC9400529

[2] Havers FP, Pham H, Taylor CA, COVID19-associated hospitalizations among vaccinated and unvaccinated adults ≥18 years—COVID-NET, 13 states, January 1, 2021–April 30, 2022. JAMA Intern Med. In press 2022. 10.1001/jamainternmed.2022.4299 PMC9459904 36074486

[3] Delahoy MJ, Whitaker M, O’Halloran A, ; COVID-NET Surveillance Team. Characteristics and maternal and birth outcomes of hospitalized pregnant women with laboratory-confirmed COVID-19—COVID-NET, 13 States, March 1–August 22, 2020. MMWR Morb Mortal Wkly Rep 2020;69:1347–54. 10.15585/mmwr.mm6938e1 32970655 PMC7727497

[4] Garg S, Kim L, Whitaker M, Hospitalization rates and characteristics of patients hospitalized with laboratory-confirmed coronavirus disease 2019—COVID-NET, 14 States, March 1–30, 2020. MMWR Morb Mortal Wkly Rep 2020;69:458–64. 10.15585/mmwr.mm6915e3 32298251 PMC7755063

[5] Link-Gelles R, Levy ME, Gaglani M, Effectiveness of 2, 3, and 4 COVID-19 mRNA vaccine doses among immunocompetent adults during periods when SARS-CoV-2 Omicron BA.1 and BA.2/BA.2.12.1 sublineages predominated—VISION Network, 10 states, December 2021–June 2022. MMWR Morb Mortal Wkly Rep 2022;71:931–9. 10.15585/mmwr.mm7129e1 35862287 PMC9310634

[6] Feikin DR, Higdon MM, Abu-Raddad LJ, Duration of effectiveness of vaccines against SARS-CoV-2 infection and COVID-19 disease: results of a systematic review and meta-regression. Lancet 2022;399:924–44. 10.1016/S0140-6736(22)00152-0 35202601 PMC8863502

